# The Evolution of the Isolation Hospital

**Published:** 1913-11-22

**Authors:** H. Franklin Parsons

**Affiliations:** late Assistant M.O. L.G.B.


### THE EVOLUTION OF THE ISOLATION HOSPITAL.

By the late H. FRANKLIN PARSONS, M.D., D.P.H., late Assistant M.O. L.G.B.

The germ of the idea of hospital provision for infectious diseases, not merely for the relief of the sufferer but also for the protection of the community, may be found in the leper houses and pest houses of bygone centuries; but it was only in the year 1866 that the power to provide hospital accommodation was explicitly given to local authorities. Long prior to this date, however, local benevolence had provided fever hospitals or, as they were frequently termed, Houses of Recovery, in many large cities. According to Dr. Murchison, these were the outcome of the fatal typhus epidemics which committed such ravages at the close of the eighteenth century, and the earliest was opened at Chester by Dr. Haygarth. The London Smallpox Hospital had, however, been established in 1745, and the Lock Hospital for venereal diseases in 1746. The London Fever Hospital was established in 1802. Except for such hospitals the only accommodation for patients suffering from infectious diseases, elsewhere than in their own homes, was in the general hospitals or in the workhouse infirmaries. In the workhouse infirmaries infectious cases were mixed with other patients, to the danger of the latter, and were nursed and attended to by pauper nurses, commonly feeble old men and women, ignorant of nursing, and too often vicious and neglectful. In the general hospitals also the infectious cases—largely typhus—were usually treated in the same wards with those of non-infectious diseases; and, indeed, many experienced physicians deemed it better to distribute the fever cases among other patients in general wards, provided that the proportion of the former was kept low, and that sufficient space and ventilation were provided, than to treat them in special wards or buildings. This view, however, was controverted by Dr. C. Murchison.

It had already been recognised that smallpox cases could not be treated along with other patients without great danger of the disease spreading; and as the provision of special isolation hospitals became more frequent, the practice of receiving other infectious cases into general hospitals gradually ceased. Enteric fever and diphtheria, however, long continued to be received into general hospitals, the infectious nature of these diseases not being so generally recognised formerly as it is now—indeed, it was only in 1889 that diphtheria became legally admissible into the hospitals of the Metropolitan Asylums Board—and a few cases of urgency, especially those requiring operation, still find their way into general hospitals. The Sanitary Act of 1866, and subsequently the Public Health Act, 1875, empowered local authorities to provide " hospitals or temporary places for the reception of the sick." The sick for whom this provision may be made are not limited to those suffering from infectious diseases, and, indeed, a few local authorities have, under special circumstances, made use of their powers to provide or contribute to hospitals for accident cases, or for those of other diseases. But public opinion in this country has generally favoured the view that the care of the non-infectious sick, in so far as it does not fall within the province of the Poor Law, is best left to private charity.

The earliest hospitals provided by the local authorities were emergency structures intended to combat some formidable epidemic disease, and were either temporary buildings hastily constructed of wood or similar material, or existing buildings converted for use as hospitals. Sometimes these were disused when the emergency had passed, but in other instances they were retained for the reception of cases of the infectious diseases of ordinary occurrence in the district, and thus formed the germs from which permanent isolation hospitals sprung. It was found, however, that, as was pointed out by Sir R. Thorne in his Report to the Local Government Board in 1882, " On the Use and Influence of Hospitals for Infectious Diseases," that hospitals thus hurriedly erected under the influence of panic had often not been ready for use until the immediate need for them was past; that they were commonly ill-planned and ill-constructed, and that the accommodation which they provided failed, almost without exception, to meet the permament requirements of the district, even when in amount it proved to be more than the district needed. In these respects they were in contrast to the hospitals which had been carefully planned and erected during non-epidemic periods, with a view of preventing epidemics by having in actual readiness means for the isolation of first cases of infectious disease.

Since the issue of Sir R. Thorne's Report the provision of isolation hospitals has become much more usual. In 1879 the replies to a circular letter issued by the Local Government Board showed that at that time, out of a total of 1,593 sanitary authorities in England and Wales, 296, or 18.6 per cent., possessed means of some sort or other for the isolation of infectious diseases; but the hospital provision was in many cases such in name only, being rarely or never used, or was available only for one disease, usually smallpox. At the end of 1892, according to a Parliamentary return issued in 1895, out of 1,653 sanitary authorities then existing in England and Wales, outside London, 631, or 38.2 per cent., had provided alone or jointly with other authorities some kind of hospital accommodation for infectious diseases.

During the seven years 1893-1899 the Local Government Board sanctioned 334 loans for hospital purposes—not all, however, relating to newly established or to different hospitals—while many other authorities provided themselves with hospital accommodation without the aid of loans, either by the erection or adaptation of buildings of their own —the accommodation thus provided being indeed often of a more or less temporary or make-shift character—or by entering into agreements for the use of hospitals for other districts.

The principle of combination of districts has received much extension since the date of Sir Richard Thorne's Report. At the time when he wrote there were a few such combinations, some under a Joint Hospital Board, established by Provisional Orders confirmed by Parliament; others under joint committees less formally constituted. Sir Richard Thorne, however, was of opinion that an isolation hospital should, if possible, be within the limits of the district for which it was erected, and that in order to be reasonably available it should, in the case of a town, be distant not much more than two miles, and in the case of a rural district, not more than four or five miles from the populous parts of the district. He did not, indeed, find that removal for a distance of some five, or even eight or ten, miles in a well-constructed ambulance over ordinarily good roads had appeared to do harm to the patients, provided their removal had been effected at an early stage of their illness, but he found that the relations and friends assented much more readily to their removal to a hospital within an easy distance than to a more distant one. But in respect to this question circumstances have much altered since Sir Richard Thorne wrote. With experience of the use of isolation hospitals, and increased public appreciation of the advantages of hospital treatment, the objection to removal of patients, even to a hospital at a distance from their homes, has in most parts of the country much diminished.

As a matter of fact it is found that the area which a hospital can serve is much larger than was formerly supposed. Again it has been found that as compared with the establishment of separate small hospitals for individual districts, the provision of a large hospital for a combined area presents distinct advantages. There is only one site to be obtained, with a larger area for selection; which is an important gain in view of the difficulty often met with in obtaining hospital sites. Then, again, the preparation of a site for hospital purposes involves expenditure on fencing, road-making, drainage, water-supply, and similar matters; these are necessary for a hospital, however small; but the work needed even for a small hospital will serve with comparatively small increase for a considerably larger one. The same remark applies also to administrative accommodation, such as quarters for nurses and other staff, laundry facilities, disinfecting apparatus, etc.—matters in respect of which the earlier isolation hospitals were but poorly equipped. The provision of these on a scale adequate for even a small hospital forms no inconsiderable part of its cost; but the same provision will serve with but little increase for a hospital containing a considerably larger number of beds. In a larger hospital containing more wards there are greater facilities for the classification of patients with different diseases, and it is easier to maintain an efficient staff than in a small hospital serving a single district only, which may often be void of patients for long periods.

The number of Joint Hospital Boards, which was thirty-two in 1892, increased to fifty-one at the end of 1900, and eighty-three in 1910, while other districts combined in a less formal manner. In the case of small districts in the neighbourhood of a large town it is usual for the former to make arrangements for the use of the hospital of the latter on terms agreed on.

Concurrent powers of hospital provision and combination of districts were given by the Isolation Hospitals Act, 1893, which placed in the hands of County Councils powers for the formation of hospital districts and promotion of isolation hospitals. In some counties, as Wiltshire, Derbyshire, and the West Riding of Yorkshire, these powers have been freely exercised, and up to the end of 1909 some seventy hospital committees had been formed by Orders of County Councils.

Smallpox stands in a different category from the other infectious diseases commonly treated in isolation hospitals. It has been found by experience that smallpox cannot be treated in the same hospital with other diseases, even in a different building, without risk of its spreading to the other patients; and it has also been found that smallpox tends to spread to persons living in the neighbourhood of hospitals in which many acute cases are under treatment. There has been much controversy as to whether this spread takes place by the agency of aerial dissemination or of personal communication, but the fact that it has often taken place is beyond question; and it has become recognised as necessary that smallpox should be treated in separate hospitals, and that these should be at an ample distance from populous localities. The advantages which have been cited in favour of combination over the independent provision of small hospitals for separate districts apply with especial force in the case of smallpox hospitals. Smallpox patients bear removal well, and people are commonly anxious to have them removed, so that with a proper ambulance it has been found practicable to convey them a distance of twenty miles. Hence a smallpox hospital may conveniently serve a larger area than one for other infectious diseases; and in some instances districts which had provided themselves with hospital accommodation for the ordinary infectious diseases separately or by smaller combinations have taken part in a larger combination for the provision of a smallpox hospital. A special advantage of combination in the case of smallpox is that as the disease is now comparatively infrequent a hospital provided for a single district would often be without patients for long periods, whereas a hospital serving many districts would be less often unoccupied, and the expense of its maintenance in readiness when empty would be shared between all the districts. In view also of its being frequently empty it is undesirable that the accommodation provided at a smallpox hospital should be on a large scale or comprise costly appliances; it may contain only a few permanent beds for the prompt isolation of the sporadic cases which occur from time to time, with space and facilities for rapid extension on the occasion of an epidemic.

